# Atomic context-conditioned protein sequence design using LigandMPNN

**DOI:** 10.1038/s41592-025-02626-1

**Published:** 2025-03-28

**Authors:** Justas Dauparas, Gyu Rie Lee, Robert Pecoraro, Linna An, Ivan Anishchenko, Cameron Glasscock, David Baker

**Affiliations:** 1 Department of Biochemistry, University of Washington, Seattle, WA USA; 2 Institute for Protein Design, University of Washington, Seattle, WA USA; 3 Howard Hughes Medical Institute, University of Washington, Seattle, WA USA; 4 Department of Physics, University of Washington, Seattle, WA USA

**Keywords:** Proteins, Biophysical chemistry, Protein design, Computational platforms and environments

## Abstract

Protein sequence design in the context of small molecules, nucleotides and metals is critical to enzyme and small-molecule binder and sensor design, but current state-of-the-art deep-learning-based sequence design methods are unable to model nonprotein atoms and molecules. Here we describe a deep-learning-based protein sequence design method called LigandMPNN that explicitly models all nonprotein components of biomolecular systems. LigandMPNN significantly outperforms Rosetta and ProteinMPNN on native backbone sequence recovery for residues interacting with small molecules (63.3% versus 50.4% and 50.5%), nucleotides (50.5% versus 35.2% and 34.0%) and metals (77.5% versus 36.0% and 40.6%). LigandMPNN generates not only sequences but also sidechain conformations to allow detailed evaluation of binding interactions. LigandMPNN has been used to design over 100 experimentally validated small-molecule and DNA-binding proteins with high affinity and high structural accuracy (as indicated by four X-ray crystal structures), and redesign of Rosetta small-molecule binder designs has increased binding affinity by as much as 100-fold. We anticipate that LigandMPNN will be widely useful for designing new binding proteins, sensors and enzymes.

## Abstract (web summary)

LigandMPNN is a deep-learning-based protein sequence design method that can explicitly model all nonprotein components of biomolecular systems.

## Main

De novo protein design enables the creation of novel proteins with new functions, such as catalysis^1^, DNA, small-molecule and metal binding, and protein-protein interactions^2–10^. De novo design is often carried out in three steps^11–14^: first, the generation of protein backbones predicted to be near optimal for carrying out the new desired function^15–19^; second, design of amino-acid sequences for each backbone to drive folding to the target structure and to make the specific interactions required for function (for example, an enzyme active site)^20–30^; and third, sequence–structure compatibility filtering using structure prediction methods^31–36^. In this Article, we focus on the second step, protein sequence design. Both physically based methods such as Rosetta^37–39^ and deep-learning-based models such as ProteinMPNN^28^, IF-ESM^29^ and others^31–36^ have been developed to solve this problem. The deep-learning-based methods outperform physically based methods in designing sequences for protein backbones, but currently available models cannot incorporate nonprotein atoms and molecules. For example, ProteinMPNN explicitly considers only protein backbone coordinates while ignoring any other atomic context, which is critical for designing enzymes, nucleic-acid-binding proteins, sensors and all other protein functions involving interactions with nonprotein atoms.

## Results

**Fig. 1:**
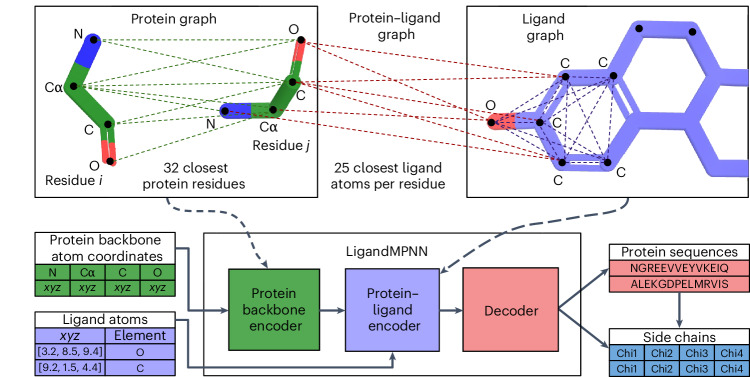
The LigandMPNN model. LigandMPNN operates on three different graphs. First, a protein-only graph with residues as nodes and 25 distances between N, Cα, C, O and virtual (inferred location based on backbone coordinates to handle the glycine case) Cβ atoms for residues *i* and *j*. Second, an intraligand graph with atoms as nodes that encodes chemical element types and distances between atoms as edges. Third, a protein–ligand graph with residues and ligand atoms as nodes and edges encoding residue *j* and ligand atom geometry. The LigandMPNN model has three neural network blocks: a protein backbone encoder, a protein–ligand encoder and a decoder. Protein sequences and sidechain torsion angles are autoregressively decoded to obtain sequence and full protein structure samples. The dotted lines show atom interactions. Metaparameter variation and ablation experiments are described in Supplementary Fig. 1a–e.

To enable the design of this wide range of protein functions, we set out to develop a deep-learning method for protein sequence design that explicitly models the full nonprotein atomic context. We sought to do this by generalizing the ProteinMPNN architecture to incorporate nonprotein atoms. As with ProteinMPNN, we treat protein residues as nodes and introduce nearest-neighbor edges based on Cα–Cα distances to define a sparse protein graph (Fig. 1); protein backbone geometry is encoded into graph edges through pairwise distances between N, Cα, C, O and Cβ atoms. These input features are then processed using three encoder layers with 128 hidden dimensions to obtain intermediate node and edge representations. We experimented with introducing two additional protein–ligand encoder layers to encode protein–ligand interactions. We reasoned that, with the backbone and ligand atoms fixed in space, only ligand atoms in the immediate neighborhood (within ~10 Å) would affect amino-acid sidechain identities and conformations because the interactions (van der Waals, electrostatic, repulsive and solvation) between ligands and sidechains are relatively short range^40^.

To transfer information from ligand atoms to protein residues, we construct a protein–ligand graph with protein residues and ligand atoms as nodes and edges between each protein residue and the closest ligand atoms. We also build a fully connected ligand graph for each protein residue with its nearest-neighbor ligand atoms as nodes; message passing between ligand atoms increases the richness of the information transferred to the protein through the ligand–protein edges. We obtained the best performance by selecting for the protein–ligand and individual residue intraligand graphs the 25 closest ligand atoms based on protein virtual Cβ and ligand atom distances (Supplementary Fig. 1a). The ligand graph nodes are initialized to one-hot-encoded chemical element types, and the ligand graph edges to the distances between the atoms (Fig. 1). The protein–ligand graph edges encode distances between N, Cα, C, O and virtual Cβ atoms and ligand atoms (Fig. 1). The protein–ligand encoder consists of two message-passing blocks that update the ligand graph representation and then the protein–ligand graph representation. The output of the protein–ligand encoder is combined with the protein encoder node representations and passed into the decoder layers. We call this combined protein–ligand sequence design model LigandMPNN.

To facilitate the design of symmetric^9,16^ and multistate proteins^10^, we use a random autoregressive decoding scheme to decode the amino-acid sequence as in the case of ProteinMPNN. With the addition of the ligand atom geometry encoding and the extra two protein–ligand encoder layers, the LigandMPNN neural network has 2.62 million parameters compared with 1.66 million ProteinMPNN parameters. Both networks are high-speed and lightweight (ProteinMPNN 0.6 s and LigandMPNN 0.9 s on a single central processing unit for 100 residues), scaling linearly with respect to the protein length. We augmented the training dataset by randomly selecting a small fraction of protein residues (2–4%) and using their sidechain atoms as context ligand atoms in addition to any small-molecule, nucleotide and metal context. Although this augmentation did not significantly increase sequence recoveries (Supplementary Fig. 1b), training in this way also enables the direct input of sidechain atom coordinates to LigandMPNN to stabilize functional sites of interest.

We also trained a sidechain packing neural network using the basic LigandMPNN architecture to predict the four sidechain torsion angles for each residue following the sequence design step. The sidechain packing model takes as input the coordinates of the protein backbone and any ligand atoms, and the amino-acid sequence, and outputs the coordinates of the protein sidechains with log-probability scores. The model predicts a mixture (three components) of circular normal distributions for the torsion angles (chi1, chi2, chi3 and chi4). For each residue, we predict three mixing coefficients, three means and three variances per chi angle. We autoregressively decompose the joint chi angle distribution by decoding all chi1 angles first, then all chi2 angles, chi3 angles and finally all chi4 angles (after the model decodes one of the chi angles, its angular value and the associated three-dimensional atom coordinates are used for further decoding).

**Fig. 2:**
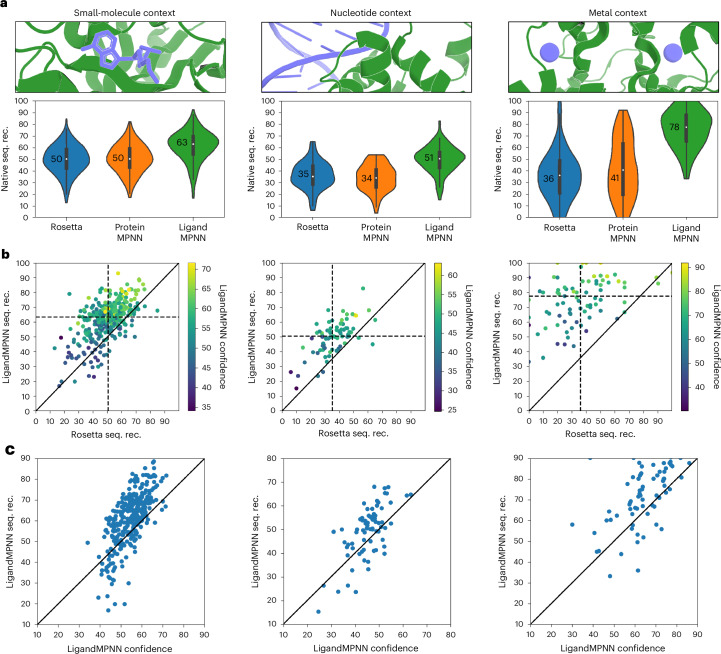
In silico evaluation of LigandMPNN sequence design. **a**, LigandMPNN has a higher recovery of native protein amino-acid identities than Rosetta and ProteinMPNN around small molecules, nucleic acids and metals. Sequence recoveries (sec. rec.) are averaged over the residues within 5.0 Å from the context atoms. **b**, LigandMPNN has higher sequence recovery around nonprotein molecules than Rosetta for most proteins. The color indicates the LigandMPNN-predicted confidence (between 0 and 100) for a given protein. The dashed lines show the mean values. **c**, Native sequence recovery correlates with LigandMPNN predicted confidence for designed sequences. One dot represents an average sequence recovery over 10 sequences for one protein for 317 small-molecule-, 74 nucleotide- and 83 metal-containing test proteins. Source data

**Fig. 3:**
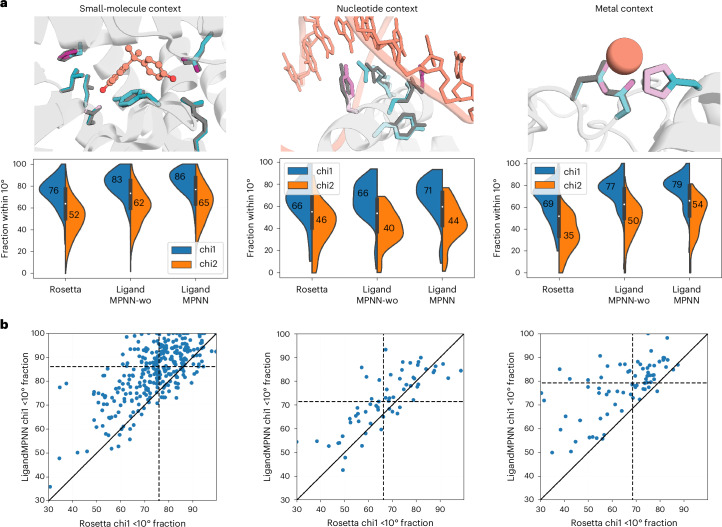
Evaluation of LigandMPNN sidechain packing accuracy. **a**, Comparison of crystal sidechain packing (gray) with LigandMPNN sidechain packing (colored sidechains by model confidence: teal is high and purple is low confidence per chi angle) for 2P7G, 1BC8 and 1E4M proteins. The context atoms are shown in orange (small molecule, DNA and zinc). LigandMPNN has higher chi1 and chi2 torsion angle recovery (fraction of residues within 10° from native) than Rosetta and LigandMPNN-wo. **b**, Per-protein comparison of chi1 fraction recovery for LigandMPNN versus Rosetta. One dot represents an average chi1 recovery over 10 sidechain packing samples for one protein for 317 small-molecule-, 67 nucleotide- and 76 metal-containing test proteins. The dashed lines show the mean values. Source data

LigandMPNN was trained on protein assemblies in the Protein Data Bank (PDB; as of 16 December 2022) determined by X-ray crystallography or cryo-electron microscopy to better than 3.5 Å resolution and with a total length of less than 6,000 residues. The train–test split was based on protein sequences clustered at a 30% sequence identity cutoff. We evaluated LigandMPNN sequence design performance on a test set of 317 protein structures containing small molecules, 74 with nucleic acids and 83 with a transition metal (Fig. 2a). For fair comparison, we retrained ProteinMPNN on the same training dataset of PDB biounits as LigandMPNN (the retrained model is referred to as ProteinMPNN in this Article), except none of the context atoms was provided during training. Protein and context atoms were noised by adding 0.1 Å standard deviation Gaussian noise to avoid protein backbone memorization^28^. We determined the native amino-acid residue sequence recovery for positions close to the ligand (with sidechain atoms within 5.0 Å of any nonprotein atoms). The median sequence recoveries (ten designed sequences per protein) near small molecules were 50.4% for Rosetta using the genpot energy function^18^, 50.4% for ProteinMPNN and 63.3% for LigandMPNN. For residues near nucleotides, median sequence recoveries were 35.2% for Rosetta^2^ (using an energy function optimized for protein–DNA interfaces), 34.0% for ProteinMPNN and 50.5% for LigandMPNN, and for residues near metals, 36.0% for Rosetta^41^, 40.6% for ProteinMPNN and 77.5% for LigandMPNN (Fig. 2a). Sequence recoveries were consistently higher for LigandMPNN over most proteins in the validation dataset (Fig. 2b; performance was correlated, probably reflecting variation in the crystal structure and the amino-acid composition of the site). LigandMPNN predicts amino-acid probability distributions and uncertainties for each residue position; the expected confidence correlates with the actual sequence recovery accuracy (Fig. 3c).

To assess the contributions to this high sustained performance, we evaluated versions in which metaparameters and features were varied or ablated (Supplementary Fig. 1a–e). Decreasing the number of context atoms per residue primarily diminished sequence recovery around nucleic acids, probably because these are larger and contain more atoms on average than small molecules and metals (Supplementary Fig. 1a). Providing sidechain atoms as additional context did not significantly affect LigandMPNN performance (Supplementary Fig. 1b). As observed for ProteinMPNN, sequence recovery is inversely proportional to the amount of Gaussian noise added to input coordinates. The baseline model was trained with 0.1 Å standard deviation noise to reduce the extent to which the native amino acid can be read out simply on the basis of the local geometry of the residue; crystal structure refinement programs introduce some memory of the native sequence into the local backbone. Training with 0.05 Å and 0.2 Å noise instead increased and decreased sequence recovery by about 2%, respectively (Supplementary Fig. 1c; when comparing performance across methods, similar levels of noising must be used). Ablating the protein–ligand and ligand graphs led to a 3% decrease in sequence recovery (Supplementary Fig. 1d). Training on sidechain context atoms only (no small molecules, nucleotides or metals) reduced sequence recovery around small molecules by 3.3% (Supplementary Fig. 1e). Finally, a model trained without chemical element types as input features had much lower sequence recovery near metals (8% difference; Supplementary Fig. 1d) but almost the same sequence recovery near small molecules and nucleic acids, suggesting that the model can to some extent infer chemical element identity from bonded geometry.

We evaluated LigandMPNN sidechain packing performance on the same dataset for residues within 5.0 Å from the context atoms. We generated ten sidechain packing examples with the fixed backbone and fixed ligand context using Rosetta, LigandMPNN and LigandMPNN without ligand context (LigandMPNN-wo in Fig. 3). The median chi1 fraction (within 10° from crystal packing) near small molecules was 76.0% for Rosetta, 83.3% for LigandMPNN-wo and 86.1% for LigandMPNN, near nucleotides 66.2%, 65.6% and 71.4% and near metals 68.6%, 76.7% and 79.3% for the three models, respectively (Fig. 3a). LigandMPNN has a higher chi1 fraction recovery compared with Rosetta on most of the test proteins (Fig. 3b), but only marginally better than LigandMPNN-wo (Supplementary Fig. 3c), suggesting that most of the information about sidechain packing is coming from the protein context rather than from the ligand context, consistent with binding site preorganization. All the models struggle to predict chi3 and chi4 angles correctly. For LigandMPNN, weighted average fractions of correctly predicted chi1, chi2, chi3 and chi4 angles for the small-molecule dataset were 84.0%, 64.0%, 28.3% and 18.7%, for Rosetta 74.5%, 50.5%, 24.1% and 8.1% and for LigandMPNN-wo 81.6%, 60.4%, 26.7% and 17.4% (Supplementary Fig. 3b). The sidechain root-mean-square deviations are similar between the different methods as shown in Supplementary Figs. 4 and 5. Comparing LigandMPNN-wo versus LigandMPNN, the biggest improvements in terms of root-mean-square deviation are obtained for glutamine (Q) in the small-molecule dataset, for arginine (R) in the nucleotide dataset and for histidine (H) in the metal context dataset (Supplementary Fig. 5), consistent with the important roles of interactions of these residues with the corresponding ligands.

**Fig. 4:**
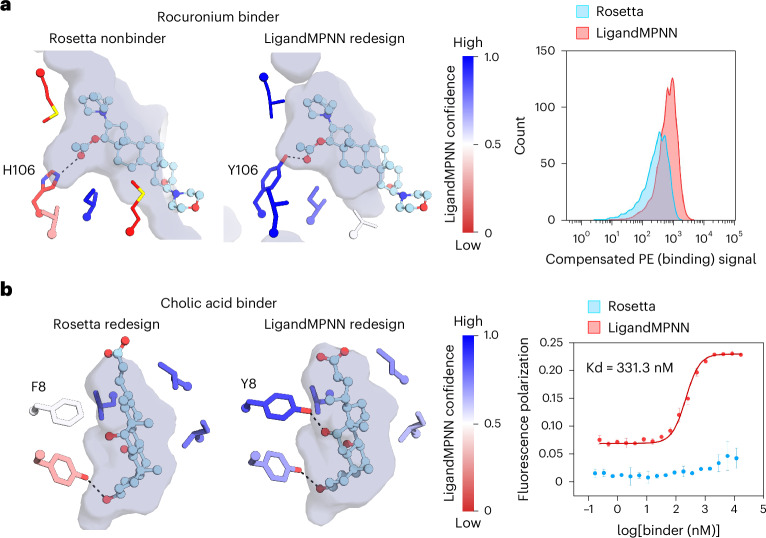
Rescue of Rosetta small-molecule binder designs using LigandMPNN. **a**,**b**, Weak or nonbinding designs made using Rosetta for rocuronium (**a**) and cholic acid (**b**) were redesigned using LigandMPNN. Left: sidechain–ligand interactions before and after redesign. The sidechains are predicted to be considerably more preorganized following redesign as indicated by the LigandMPNN amino-acid probabilities, which are colored from red (0) to blue (1). Sidechain atoms except for carbon are color-coded (O, red; N, blue; S, yellow). Right: experimental measurement of binding. In **a**, flow cytometry of yeast is shown, with the designs following incubation with 1 μM biotinylated rocuronium and streptavidin phycoerythrin. In **b**, fluorescence polarization measurements of binding to cholic acid–fluorescein isothiocyanate are shown. The error bars show the mean and standard deviations for three LigandMPNN and two Rosetta measurements. Source data

We tested the capability of LigandMPNN to design binding sites for small molecules starting from previously characterized designs generated using Rosetta that either bound weakly or not at all to their intended targets: the muscle relaxant rocuronium, for which no binding was previously observed (Fig. 4a) and the primary bile acid cholic acid (Fig. 4b) for which binding was very weak^3,4^. LigandMPNN was used to generate sequences around the ligands using the backbone and ligand coordinates as input; these retain and/or introduce new sidechain–ligand hydrogen bonding interactions. LigandMPNN redesigns either rescued binding (Fig. 4a and Supplementary Fig. 6) or improved the binding affinity (Fig. 4b). A further example with cholic acid is described in ref. ^4^, where, starting from the crystal structure of a previously designed complex, LigandMPNN increased binding affinity 100-fold. As with the many other design successes with LigandMPNN (see below), these results indicate significant generalization beyond the PDB training set: there were no rocuronium-binding protein complex structures in the PDB training set, and the cholic-acid-binding protein in the PDB that is closest to our cholic-acid-binding design (PDB: 6JY3) has a quite different structure (template modeling score 0.59) with a totally different ligand-binding location (Supplementary Fig. 7).

## Discussion

The deep-learning-based LigandMPNN is superior to the physically based Rosetta for designing amino acids to interact with nonprotein molecules. It is about 250 times faster (because the expensive Monte Carlo optimization over sidechain identities and compositions is completely bypassed), and the recoveries of native amino-acid identities and conformations around ligands are consistently higher. The method is also easier to use because no expert customizations are required for new ligands (unlike Rosetta and other physically based methods that can require new energy function or force field parameters for new compounds). At the outset, we were unsure whether the accuracy of ProteinMPNN could extend to protein–ligand systems given the small amount of available training data, but our results suggest that, for the vast majority of ligands, there are sufficient data. Nevertheless, we suggest some care in using LigandMPNN for designing binders to compounds containing elements occurring rarely or not at all in the PDB (in the latter case it is necessary to map to the most closely occurring element). Hybridization of the physically based and deep-learning-based approaches may provide a better solution to the amino-acid and sidechain optimization problems in the low-data regime.

LigandMPNN has already been extensively used for designing interactions of proteins with nucleic acids and small molecules, and these studies provide considerable additional experimental validation of the method. In these studies, LigandMPNN was either used as a drop-in replacement for Rosetta sequence design retaining the backbone relaxation of RosettaFastDesign^38,42^, or used independently without backbone relaxation. Glasscock et al.^2^ developed a computational method for designing small sequence-specific DNA-binding proteins that recognize specific target sequences through interactions with bases in the major groove that uses LigandMPNN to design the protein–DNA interface. The crystal structure of a DNA-binding protein designed with LigandMPNN recapitulated the design model closely (deposited to the Research Collaboratory for Structural Bioinformatics Protein Data Bank as PDB ID 8TAC). Lee et al.^3^, An et al.^4^ and Krishna et al.^5^ used LigandMPNN to design small-molecule-binding proteins with scaffolds generated by deep-learning- and Rosetta-based methods. Iterative sequence design with LigandMPNN resulted in nanomolar-to-micromolar binders for the 17α-hydroxyprogesterone, apixaban and SN-38 with NTF2-family scaffolds^3^, nanomolar binders for cholic acid, methotrexate and thyroxine^4^ in pseudocyclic scaffolds, and binders for digoxigenin, heme and bilin in RFdiffusion_allatom-generated scaffolds^5^. In total, more than 100 protein–DNA binding interfaces and protein–small-molecule binding interfaces designed using LigandMPNN have been experimentally demonstrated to bind to their targets, and 5 co-crystal structures have been solved that in each case are very close to the computational design models^3–5^. This extensive biochemical and structural validation provides strong support for the power of the approach.

As with ProteinMPNN, we anticipate that LigandMPNN will be widely useful in protein design, enabling the creation of a new generation of small-molecule-binding proteins, sensors and enzymes. To this end, we have made the code available via GitHub at https://github.com/dauparas/LigandMPNN.

## Methods

### Methods for training LigandMPNN for sequence design

#### Training data

LigandMPNN was trained on a dataset similar to ProteinMPNN^28^. We used protein assemblies in the PDB (as of 16 December 2022) determined by X-ray crystallography or cryo-electron microscopy to better than 3.5 Å resolution and with fewer than 6,000 residues. We parsed all residues present in the PDBs except [‘HOH’, ‘NA’, ‘CL’, ‘K’, ‘BR’]. Protein sequences were clustered at 30% sequence identity cutoff using mmseqs2 (ref. ^43^). We held out a nonoverlapping subset of proteins that have small-molecule contexts (a total of 317), nucleotide contexts (a total of 74) and metal contexts (a total of 83).

#### Optimizer and loss function

For optimization, we used Adam with beta1 of 0.9, beta2 of 0.98 and epsilon of 1e-9, the same as for ProteinMPNN. Models were trained with a batch size of 6,000 tokens, automatic mixed precision and gradient checkpointing on a single NVIDIA A100 graphics processing unit for 300,000 optimizer steps. We used categorical cross entropy for the loss function following the ProteinMPNN paper^28^.

#### Input featurization and model architecture

We used the same input features as in the ProteinMPNN paper for the protein part. For the atomic context input features, we used one-hot-encoded chemical element types as node features for the ligand graph and the radial basis function-encoded distances between the context atoms as edges for the ligand graph. To encode the interaction between protein-context atoms, we used distances between N, Cα, C, O and virtual Cβ atoms and context atoms. In addition, we added angle-based sin/cos features describing context atoms in the frame of N–Cα–C atoms.

We used the same MPNN architecture as used in ProteinMPNN for the encoder, decoder and protein–ligand encoder blocks. Encoder and decoder blocks work on protein nodes and edges, that is, mapping vertices [*N*] and edges [*N*, *K*] to updated vertices [*N*] and edges [*N*, *K*] where *N* is the number of residues and *K* is the number of direct neighbors per residue. We choose *M* context atoms per residue resulting in [*N*, *M*] protein–atom interactions. The ligand graph blocks map vertices of size [*N*, *M*] and edges of size [*N*, *M*, *M*] (fully connected context atoms) to updated vertices [*N*, *M*]. The updated [*N*, *M*] representation is used in the protein–ligand graph to map vertices [*N*] and edges [*N*, *M*] into updated vertices [*N*]. For more details, refer to the LigandMPNN code.

#### Model algorithms

We provide a list of algorithms and model layers used by the LigandMPNN model. The model is based on the autoregressive encoder-decoder architecture. Algorithm 10 describes how the input features such as protein atom coordinates (*X*), ligand coordinates (*Y*), ligand mask (*Y*_m), and ligand atom types (*Y*_t) are converted into the input features. Protein and ligand geometric features are encoded using the algorithm 11, and it returns final protein node and edge features. Finally, algorithm 12 decodes protein sequence by predicting log probabilities for all amino acids. During the inference, we sample from these probabilities with some temperate (*T*) (algorithm 13) and iteratively run algorithm 12 to populate the designed sequence (*S*).

Notation:

*X* ∈ ℝ^*L*×4×3^- protein backbone coordinates for N, Cα, C and O atoms with *L* residues

*Y* ∈ ℝ^*L*×M×3^- coordinates of the closest *M* ligand atoms from the virtual Cβ atom in the protein

*Y*_m ∈ ℝ^*L*×*M*^- ligand atom mask

*Y*_t ∈ ℝ^*L*×*M*^- ligand atom type

##### Algorithm 1

Linear layer

**def** Linear(x ∈ **ℝ**^*n*^; W ∈ **ℝ**^*m×n*^, b ∈ **ℝ**^*m*^):

1: x ← Wx+b, x ∈ **ℝ**^*m*^

2: **return** x

##### Algorithm 2

Non-linear layer^44^

**def** GELU(x ∈ **ℝ**^*n*^):

1: x ← 0.5⋅x⋅(1+tanh(2/π⋅(x + 0.044715⋅x^*3*^))), x ∈ **ℝ**^*n*^

2: **return** x

##### Algorithm 3

Normalization layer

**def** LayerNorm(x ∈ **ℝ**^*n*^; γ ∈ **ℝ**^*n*^, β ∈ **ℝ**^*n*^):

1: μ = E[x]=(x_*1*_ + x_*2*_ + …+x_*n*_)/n, μ ∈ **ℝ**^*n*^

2: σ^*2*^ = E[(x-μ)^*2*^], σ^*2*^ ∈ **ℝ**^*n*^

3: x ← γ⋅(x-μ)/σ + β, x ∈ **ℝ**^*n*^

4: **return** x

##### Algorithm 4

Dropout layer

**def** Dropout(x ∈ **ℝ**^*n*^; p ∈ **ℝ**, training: bool):

1: if training:

2:  mask = Binomial[1-p](x.shape), mask ∈ **ℝ**^*n*^

3:  x ← mask⋅x/(1-p), x ∈ **ℝ**^*n*^

4:  **return** x

5: else:

6:  **return** x

##### Algorithm 5

Position wise feed-forward

**def** PositionWiseFeedForward (v_*i*_ ∈ **ℝ**^*n*^; *n* = *128, m* = *512*):

1: v_*i*_ ← Linear[n,m](v_*i*_), v_*i*_ ∈ **ℝ**^*m*^

2: v_*i*_ ← GELU(v_*i*_), v_*i*_ ∈ **ℝ**^*m*^

3: v_*i*_ ← Linear[m,n](v_*i*_), v_*i*_ ∈ **ℝ**^*n*^

4: **return** v_*i*_

##### Algorithm 6

Positional encoding layer

**def** PositionalEncodings(offset ∈ **ℝ**^*L×K*^, mask ∈ **ℝ**^*L×K*^; *n* = *16, max_offset* = *32*):

  #offset - protein residue to residue distances for all chains

  #mask - mask if two residues are from the same chain

   #n - number of dimensions to embed the offset to

  #max_offset - maximum distance between two residues

1: d = mask⋅clip[0, 2⋅*max_offset*](offset + *max_offset)*, d ∈ **ℝ**^*L×L*^

2: f = (1-mask)⋅(2⋅*max_offset* + 1), f ∈ **ℝ**^*L×L*^

3: g = d + f, g ∈ **ℝ**^*L×L*^

4: g_one_hot = one_hot[2⋅*max_offset* + 2](g), g_one_hot ∈ **ℝ**^*L×L×2*^⋅^*max_offset+2*^

5: e ← Linear[2⋅*max_offset* + 2,n](g_one_hot), e ∈ **ℝ**^*L×L×n*^

6: **return** e

##### Algorithm 7

Encoder Layer

**def** EncLayer(v ∈ **ℝ**^*L×n*^, e ∈ **ℝ**^*L×K×n*^, e_idx ∈ **ℝ**^*L×K*^*; n* = *128, m* = *128, p* = *0.1, s* = *30.0*):

  #v - vertex embedding for L residues

  #e - edge embedding for L residues with K neighbors per residue

  #e_idx - integers specifying protein residue neighbor positions

  #n - input dimension

  #m - hidden dimension

  #p - dropout probability

  #s - scaling factor

1: q_*ij*_ = concatenate[e_idx_*ij*_](v_*i*_, v_*j*_, e_*ij*_), q ∈ **ℝ**^*L×K×3⋅n*^, q_*ij*_ ∈ **ℝ**^*3⋅n*^,

2: q_*ij*_ ← GELU{Linear[3n,m](q_*ij*_)}, q_*ij*_ ∈ **ℝ**^*m*^,

3: q_*ij*_ ← GELU{Linear[m,m](q_*ij*_)}, q_*ij*_ ∈ **ℝ**^*m*^,

4: q_*ij*_ ← Linear[m,m](q_*ij*_), q_*ij*_ ∈ **ℝ**^*m*^,

5: dh_*i*_ ← Σ_*j*_ q_*ij*_/s, dh_*i*_ ∈ **ℝ**^*m*^,

6: v_*i*_ ← LayerNorm{v_*i*_+Dropout[p](dh_*i*_)}, v_*i*_ ∈ **ℝ**^*m*^,

7: q_*ij*_ = concatenate[e_idx_*ij*_](v_*i*_, v_*j*_, e_*ij*_), q ∈ **ℝ**^*L×K×3⋅n*^, q_*ij*_ ∈ **ℝ**^*3⋅n*^,

8: q_*ij*_ ← GELU{Linear[3n,m](q_*ij*_)}, q_*ij*_ ∈ **ℝ**^*m*^,

9: q_*ij*_ ← GELU{Linear[m,m](q_*ij*_)}, q_*ij*_ ∈ **ℝ**^*m*^,

10: q_*ij*_ ← Linear[m,m](q_*ij*_), q_*ij*_ ∈ **ℝ**^*m*^,

11: e_*ij*_ ← LayerNorm{e_*ij*_+Dropout[p](q_*ij*_)}, v_*i*_ ∈ **ℝ**^*m*^,

12: **return** v, e

##### Algorithm 8

Decoder Layer

**def** DecLayer(v ∈ **ℝ**^*L×n*^, e ∈ **ℝ**^*L×K×2n*^*; n* = *128, m* = *128, p* = *0.1, s* = *30.0*):

  #v - vertex embedding for L residues

  #e - edge embedding for L residues with K neighbors

  #n - input dimension

   #m - hidden dimension

  #p - dropout probability

  #s - scaling factor

1: q_*ij*_ = concatenate(v_*i*_, e_*ij*_), q ∈ **ℝ**^*L×K×3⋅n*^, q_*ij*_ ∈ **ℝ**^*3⋅n*^

2: q_*ij*_ ← GELU{Linear[3n,m](q_*ij*_)}, q_*ij*_ ∈ **ℝ**^*m*^,

3: q_*ij*_ ← GELU{Linear[m,m](q_*ij*_)}, q_*ij*_ ∈ **ℝ**^*m*^,

4: q_*ij*_ ← Linear[m,m](q_*ij*_), q_*ij*_ ∈ **ℝ**^*m*^,

5: dh_*i*_ ← Σ_*j*_ q_*ij*_/s, dh_*i*_ ∈ **ℝ**^*m*^,

6: v_*i*_ ← LayerNorm{v_*i*_+Dropout[p](dh_*i*_)}, v_*i*_ ∈ **ℝ**^*m*^,

7: **return** v

##### Algorithm 9

Context Decoder Layer

**def** DecLayerJ(v ∈ **ℝ**^*L×M×n*^, e ∈ **ℝ**^*L×M×M×2n*^*; n* = *128, m* = *128, p* = *0.1, s* = *30.0*):

  #v - vertex embedding for L residues with M atoms per residue

  #e - edge for L residues with M atoms and M neighbors per atom

  #n - input dimension

  #m - hidden dimension

  #p - dropout probability

  #s - scaling factor

1: q_*ijk*_ = concatenate(v_*ij*_, e_*ijk*_), q ∈ **ℝ**^*L×M×M×3⋅n*^, q_*ijk*_ ∈ **ℝ**^*3⋅n*^,

2: q_*ijk*_ ← GELU{Linear[3n,m](q_*ijk*_)}, q_*ijk*_ ∈ **ℝ**^*m*^,

3: q_*ijk*_ ← GELU{Linear[m,m](q_*ijk*_)}, q_*ijk*_ ∈ **ℝ**^*m*^,

4: q_*ijk*_ ← Linear[m,m](q_*ijk*_), q_*ijk*_ ∈ **ℝ**^*m*^,

5: dh_*ij*_ ← Σ_*k*_ q_*ijk*_/s, dh_*ij*_ ∈ **ℝ**^*m*^,

6: v_*ij*_ ← LayerNorm{v_*ij*_+Dropout[p](dh_*ij*_)}, v_*i*_ ∈ **ℝ**^*m*^,

7: **return** v

##### Algorithm 10

Protein and ligand featurization

**def** ProteinFeaturesLigand(Y ∈ **ℝ**^*L×M×3*^, Y_m ∈ **ℝ**^*L×M*^, Y_t ∈ **ℝ**^*L×M*^, X ∈ **ℝ**^*L×4×3*^, R_idx ∈ **ℝ**^*L*^, chain_labels ∈ **ℝ**^*L*^; noise_level = 0.1, K = 32, m = 128, r = 16):

  #Y, Y_m, Y_t - ligand atom coordinates, mask, and chemical atom type

  #X - protein coordinates for N, Cα, C, O atoms in this order

  #R_idx - protein residue indices

  #chain labels - integer labels for protein chains

  #noise_level - standard deviation of Gaussian noise

  #K - number of nearest Cα neighbors for protein

  #m - hidden dimension size

  #r - radial basis function number

1: X ← X + noise_level⋅GaussianNoise(X.shape), X ∈ **ℝ**^*L×4×3*^,

2: Y ← Y + noise_level⋅GaussianNoise(Y.shape), X ∈ **ℝ**^*L×M×3*^,

3: Cβ = = -0.5827⋅[(Cα-N)^(C-Cα)] + 0.5680⋅(Cα-N) - 0.5407⋅(C-Cα) + Cα, N, Cα, C, Cβ ∈ **ℝ**^*L×3*^,

4: **e_idx** = top_k[K](||Cα_*i*_-Cα_*j*_||_*2*_), e_idx ∈ **ℝ**^*L×K*^,

5: rbf = []

6: for a in [N, Cα, C, O Cβ]:

7:  for b in [N, Cα, C, O Cβ]:

8:   rbf_tmp = rbf_f{get_edges[e_idx](||a_*i*_-b_*j*_||_*2*_)}, rbf_tmp ∈ **ℝ**^*L×K×r*^,

9:   rbf.append(rbf_tmp)

10: rbf ← concatenate(rbf), rbf ∈ **ℝ**^*L×K×25⋅r*^,

11: offset = get_edges[e_idx](R_idx_*i*_-R_idx_*j*_), offset ∈ **ℝ**^*L×K*^,

12: offset_m = get_edges[e_idx](chain_labels_*i*_-chain_labels_*j*_ = =0), offset_m ∈ **ℝ**^*L×K*^,

13: pos_enc = PositionalEncodings(offset, offset_m), pos_enc ∈ **ℝ**^*L×K×r*^

14: **e** ← LayerNorm{Linear[r + 25⋅r,m](concat[pos_enc, rbf])}, e∈ **ℝ**^*L×K×m*^

15: Y_t_g = chemical_group(Y_t), Y_t_g∈ **ℝ**^*L×M*^

16: Y_t_p = chemical_period(Y_t), Y_t_p∈ **ℝ**^*L×M*^

17: Y_t_1hot = Linear[64,147](onehot[concat(Y_t, Y_t_g, Y_t_p)]), Y_t_1hot∈ **ℝ**^*L×M×64*^

18: rbf_N_Y = rbf_f{||N-Y||_*2*_}, rbf_N_Y∈ **ℝ**^*L×M×r*^

19: rbf_Cα_Y = rbf_f{||Cα-Y||_*2*_}, rbf_Cα_Y ∈ **ℝ**^*L×M×r*^

20: rbf_C_Y = rbf_f{||C-Y||_*2*_}, rbf_C_Y ∈ **ℝ**^*L×M×r*^

21: rbf_O_Y = rbf_f{||O-Y||_*2*_}, rbf_O_Y∈ **ℝ**^*L×M×r*^

22: rbf_Cβ_Y = rbf_f{||Cβ-Y||_*2*_}, rbf_Cβ_Y∈ **ℝ**^*L×M×r*^

23: rbf_Y = concat(rbf_N_Y, rbf_Cα_Y, rbf_C_Y, rbf_O_Y,rbf_Cβ_Y), rbf_Y∈ **ℝ**^*L×M×5⋅r*^

24: angles_Y = make_angle_features(N, Cα, C, Y), angles_Y∈ **ℝ**^*L×M×4*^

25: v = concat(rbf_Y, Y_t_1hot, angles_Y), v∈ **ℝ**^*L×M×4*^

26: **v** ← LayerNorm{Linear[5⋅r + 64 + 4,m](v)}, v∈ **ℝ**^*L×M×m*^

27: Y_edges = rbf_f{||Y_*i*_-Y_*j*_||_*2*_}, Y_edges∈ **ℝ**^*L×M×M×r*^

28: **Y_edges** ← LayerNorm{Linear[r,m](Y_edges)}, Y_edges∈ **ℝ**^*L×M×M×m*^

29: **Y_nodes** = LayerNorm{Linear[147,m](onehot[concat(Y_t, Y_t_g, Y_t_p)])}, Y_nodes∈ **ℝ**^*L×M×m*^

30: **return v,** **e,** **e_idx,** **Y_nodes,** **Y_edges**

##### Algorithm 11

LigandMPNN encode function

**def** LigandMPNN_encode(Y ∈ **ℝ**^*L×M×3*^, Y_m ∈ **ℝ**^*L×M*^, Y_t ∈ **ℝ**^*L×M*^, X ∈ **ℝ**^*L×4×3*^, R_idx ∈ **ℝ**^*L*^, chain_labels ∈ **ℝ**^*L*^; num_layers=3, c_num_layers=2, m = 128):

1: v_y, e, **e_idx**, Y_nodes, Y_edges = ProteinFeaturesLigand(Y, Y_m, Y_t, X, R_idx, chain_labels)

2: v_y = Linear[m,m](v_y), v_y ∈ **ℝ**^*L×m*^,

3: v = zeros(L, m), v ∈ **ℝ**^*L×m*^,

4: for i in range(num_layers):

5:   v, **e** ← EncLayer(v, e, e_idx), v ∈ **ℝ**^*L×m*^, e ∈ **ℝ**^*L×K×m*^

6: v_c = Linear[m,m](v), v_c ∈ **ℝ**^*L×m*^,

7: Y_m_edges = Y_m_*i*_⋅Y_m_*j*_, Y_edges ∈ **ℝ**^*L×M×M*^,

8: Y_nodes = Linear[m,m](Y_nodes), Y_nodes ∈ **ℝ**^*L×M×m*^,

9: Y_edges = Linear[m,m](Y_edges), Y_edges ∈ **ℝ**^*L×M×M×m*^,

10: for i in range(c_num_layers):

11:   Y_nodes ← DecLayerJ(Y_nodes, Y_edges, Y_m, Y_m_edges) #atom graph

12:   Y_nodes_c = concat(v_y, Y_nodes)

13:   v_c ← DecLayer(v_c, Y_nodes_c, mask, Y_m) #protein graph

14: v_c ← Linear[m,m](v_c)

14: **v** ← v + LayerNorm[Dropout[p]](v_c)

15: **return v, e, e_idx**

##### Algorithm 12

LigandMPNN decode function

**def** LigandMPNN_decode(S ∈ **ℝ**^*L*^, Y_m ∈ **ℝ**^*L×M*^, Y_t ∈ **ℝ**^*L×M*^, X ∈ **ℝ**^*L×4×3*^, R_idx ∈ **ℝ**^*L*^, chain_labels ∈ **ℝ**^*L*^, decoding_order ∈ **ℝ**^*L*^; num_layers=3, m = 128):

1: h_V, e, e_idx = LigandMPNN_encode(Y, Y_m, Y_t, X, R_idx, chain_labels)

2: causal_mask = upper_triangular[decoding_order](L,L)

3: h_S = Linear[21,m](onehot(S)), h_S ∈ **ℝ**^*L×m*^,

4: h_ES = concat(h_S, e, e_idx), h_ES ∈ **ℝ**^*L×K×2m*^,

5: h_EX_encoder = concat(zeros(h_S), e, e_idx), h_EX_encoder ∈ **ℝ**^*L×K×2m*^,

6: h_EXV_encoder = concat(h_V, h_EX_encoder, e_idx), h_EXV_encoder ∈ **ℝ**^*L×K×3m*^,

7: h_EXV_encoder_fw =(1-causal_mask)⋅h_EXV_encoder

8:  for i in range(num_layers):

9:  h_ESV = conat(h_V, h_ES, e_idx)

10:  h_ESV ← causal_mask⋅h_ESV + h_EXV_encoder_fw

11:  h_V ← DecLayer(h_V, h_ESV)

12: logits = Linear[m,21](h_V), logits ∈ **ℝ**^*L×21*^,

13: **log_probs** = log_softmax(logits)

14: **return logits,** **log_probs**

##### Algorithm 13

Amino-acid sampling with temperature

**def** sampling(logits∈ **ℝ**^*21*^, T∈ **ℝ**, bias∈ **ℝ**^*21*^):

1: p = softmax((logits+bias)/T)

2: S = categorical_sample(p)

3: **return S**

##### Algorithm 14

Outline of LigandMPNN sidechain decode function

**def** LigandMPNN_sc_decode(Y_m ∈ **ℝ**^*L×M*^, Y_t ∈ **ℝ**^*L×M*^, X ∈ **ℝ**^*L×14×3*^, R_idx ∈ **ℝ**^*L*^, chain_labels ∈ **ℝ**^*L*^, decoding_order ∈ **ℝ**^*L*^; num_layers=3, m = 128):

1: h_V_enc, h_E_enc, e_idx = LigandMPNN_encode(Y, Y_m, Y_t, X, R_idx, chain_labels)

2: h_V_dec, h_E_dec = LigandMPNN_encode(Y, Y_m, Y_t, X, R_idx, chain_labels)

3: causal_mask = upper_triangular[decoding_order](L,L)

4: h_EV_encoder = concat(h_V_enc, h_E_enc, e_idx)

5: h_E_encoder_fw =(1-causal_mask)⋅h_EV_encoder

6: h_EV_decoder = concat(h_V_dec, h_E_dec, e_idx)

7: h_V = h_V_enc

8: for i in range(num_layers):

9:  ▓h_EV = conat(h_V, h_E_decoder, e_idx)

10:  ▓h_ECV ← causal_mask⋅h_EV + h_E_encoder_fw

11:  ▓h_V ← DecLayer(h_V, h_ECV)

12: torsions = Linear[m,4⋅3⋅3](h_V).reshape(L,4,3,3), torsions ∈ **ℝ**^*L×4×3×3*^,

13: mean = torsions[…,0], mean ∈ **ℝ**^*L×4×3*^,

14: concentration = 0.1 + softplus(torsions[…,1]), concentration ∈ **ℝ**^*L×4×3*^

15: mix_logits =torsions[…,2], mix_logits ∈ **ℝ**^*L×4×3*^

16: **predicted_distribution** = VonMisesMixture(mean, concentration, mix_logits)

17: **return predicted_distribution**

ProteinMPNN and LigandMPNN share the idea of using autoregressive sequence decoding with a sparse residue graph with ref. ^21^. However, there are many differences between the models. First, ProteinMPNN is trained on biological protein assemblies, and LigandMPNN on the biological protein assemblies with small molecules, nucleotides, metals and other atoms in the PDB, whereas ref. ^21^ was trained on single chains only. Second, we wanted our models to work well with novel protein backbones as opposed to crystal backbones, and for this reason, we added Gaussian noise to all the protein and other atom coordinates to blur out fine-scale details that would not be available during the design. Furthermore, we innovated by using a random autoregressive decoding scheme that fits more naturally protein sequences as opposed to left-to-right decoding used in language models and ref. ^21^. Also, we simplified input geometric features by keeping only distances between N, Cα, C, O and inferred Cβ atoms and added positional encodings that allowed us to design multiple protein chains at the same time, as opposed to using backbone local angles as in ref. ^21^. Both ProteinMPNN and LigandMPNN can design symmetric and multistate proteins by choosing an appropriate decoding order and averaging out predicted probabilities. Also, we added expressivity to our MPNN encoder layers, allowing both graph nodes and edges to be updated. LigandMPNN further builds on top of ProteinMPNN by incorporating local atomic context into the protein residue local environment using invariant features. We pass messages between protein residues and context atoms to encode possible sequence combinations. Finally, LigandMPNN can also predict with uncertainty multiple sidechain packing combinations of a newly designed sequence near nucleotides, metals and small molecules, which can help designers to choose sequences that make desired interactions with the ligand of interest. LigandMPNN can also take sidechain conformations as an input, which allows the design sequence to stabilize given ligand and selected protein sidechains.

Algorithms 1, 2, 3, 4 and 5 are commonly used in many machine learning models. Algorithms 6, 7, 8 and 13 were used in the ProteinMPNN model. Algorithms 9, 10, 11, 12 and 14 are novel and specific to LigandMPNN.

### Reporting summary

Further information on research design is available in the Nature Portfolio Reporting Summary linked to this article.

## Online content

Any methods, additional references, Nature Portfolio reporting summaries, source data, extended data, supplementary information, acknowledgements, peer review information; details of author contributions and competing interests; and statements of data and code availability are available at 10.1038/s41592-025-02626-1.

## Supplementary information

Supplementary Information Supplementary Figs. 1–8.

Reporting Summary

## Source data

Source Data Fig. 2 Sequence design raw data.

Source Data Fig. 3 Sidechain design raw data.

Source Data Fig. 4 Fluorescence polarization raw data.

## Data Availability

All data are available in the Article or its Supplementary Information. PDB structures used for training were obtained from RCSB. The following PDB IDs were used in the Article: 8VEI, 8BEJ, 8VEZ, 8VFQ, 8TAC, 6JY3, 2P7G, 1BC8 and 1E4M. (https://www.rcsb.org/docs/programmatic-access/file-download-services). Source data are provided with this paper.

## Code availability

The LigandMPNN code is available via GitHub at https://github.com/dauparas/LigandMPNN. The neural network was developed with PyTorch 1.11.0, cuda 11.1, NumPy v1.21.5, Matplotlib v3.5.1 and Python v3.9.12. MMseqs2 version 13-45111+ds-2 was used to cluster PDB chains, and mmcif vesion 0.84 (https://pypi.org/project/mmcif/) and rdkit version 2022.03.2 were used to parse PDB files. The flow cytometry data were analyzed using the software FlowJo v10.9.0.
