# Supplementary material for: Atomic context-conditioned protein sequence design using LigandMPNN
Source: Nat Methods. 2025 Mar 28;22(4):717–23. doi: 10.1038/s41592-025-02626-1 (PMC11978504; doi:10.1038/s41592-025-02626-1)
Supplement: Supplementary file 1 — Supplementary Figs. 1–8. [file 41592_2025_2626_MOESM1_ESM.pdf]

nature methods

Supplementary information https://doi.org/10.1038/s41592-025-02626-1

Atomic context-conditioned protein sequence design using LigandMPNN

In the format provided by the authors and unedited

Supplementary Information

Ablation studies

We trained several LigandMPNN variants to understand dependencies on hyperparameters (S1A-E). All sequence recoveries are reported with respect to the baseline model which was noised with 0.1 Å standard deviation, hidden dimension was 128, 3 encoder layers, 2 protein-context atom encoder layers, 2 context atom encoder layers, and 3 decoder layers, 25 context atoms per residue, 2% randomly sampled sidechains used as additional context atoms, all sequences were designed using the sampling temperature of 0.1. Firstly, we trained LigandMPNN with 2, 4, 8, 16, 25, and 32 context atoms per residue (Figure S1A). We see that sequence recoveries increase with more context atoms given but saturate at about 8-16 atoms. The most noticeable effect is on the nucleotide context residues. We also varied the fraction of sidechains used as a context, which were randomly chosen during training (Figure S1B). There is a small increase in sequence recovery for nucleotide context, but more importantly, this allows the use of sidechain atoms during the inference for fixed active site design examples. In practice, we do not know the exact backbone and context atom positions when designing sequences. As in ProteinMPNN (*1*), we added Gaussian noise to all input coordinates to avoid backbone memorization. Sequence recovery goes down for the models trained with larger amounts of noise acting as regularization (Figure S1C). For ablations (Figure S1D), we trained models without Protein-Ligand and Ligand graphs by passing protein-context atom features flattened as vertex features into the encoder. We saw about a 3% reduction in sequence recovery. Removing just the Ligand graph and keeping the Protein-Ligand graph results in about a 1% decrease. Training without chemical element types has the most effect on the residues near metal ions. However, only a marginal difference for small molecule and nucleotide context suggests that the model can infer chemical elements from the geometry. Finally, we trained LigandMPNN only using protein sidechain atoms as context (Figure S1E). Sequence recoveries near nucleotide and metal context went significantly down, however, we observed only about a 3-4% decrease in sequence recovery near small molecules, even though the model has never been trained on any small molecule data. This suggests that the model can generalize from sidechain to small-molecule contexts due to the similarity in carbon, oxygen, and nitrogen chemistry, and volume exclusion.

Sequence bias

For all three different datasets (small molecule, nucleotide, and metal), we plotted 1D (PSSM) and 2D (confusion matrix) sequence biases for Rosetta and LigandMPNN designs for the residues within 5.0 Å from the context atoms (Figure S2A-C). We noticed that when LigandMPNN is uncertain about what amino acid to predict, it predicts the most likely amino acids in these contexts. For example, it predicted a higher number of lysines near nucleotides and a slightly higher number of histidines near metals (Figure S2B). We also noted that sequence recoveries are very much correlated between ProteinMPNN and LigandMPNN for small molecule and nucleotide contexts suggesting limited information coming from the backbone. There is almost no correlation near metal context, suggesting that knowing the placement of metal ions provides very useful information to locally determine protein sequence (Figure S2C).

Sidechain modeling

We used the same architecture as for the sequence design to predict sidechain conformations. In this case, the model predicts a mixture (three components) of circular normal distributions for torsion angles chi1, chi2, chi3, and chi4. The objective was to maximize the log probability of training examples. We used the same training dataset as for sequence design with a 3.5 Å resolution cutoff. Training with higher-resolution cutoff (e.g. 2-2.5 Å) structures might improve high-accuracy sidechain modeling, but it does result in a much smaller number of nucleotide-protein structures available for training, leading to worse performance for residue near different contexts. We trained a fully autoregressive model that factorizes a joint residue-chi angle distribution into conditional distributions. Since the chi1 angles have the lowest variance, we autoregressively predict all chi1 angles first, and then autoregressively all chi2, chi3, and chi4 angles. The model predicts a chi angle distribution from which we sample a particular chi angle and pass its sin/cos values as well as 3D coordinates of corresponding atoms as an input for the next prediction. Figure S3 compares LigandMPNN-wo and Rosetta versus LigandMPNN using a 10° chi angle cutoff for residues within 5.0 Å from the context atoms. If chi1 is more than 10° away from the ground truth, then chi2, chi3, and chi4 are considered to be incorrect. Figures S4 and S5 show RMSD deviations of sidechain atoms (all sidechain atoms excluding N, Cα, C, O, and Cβ).

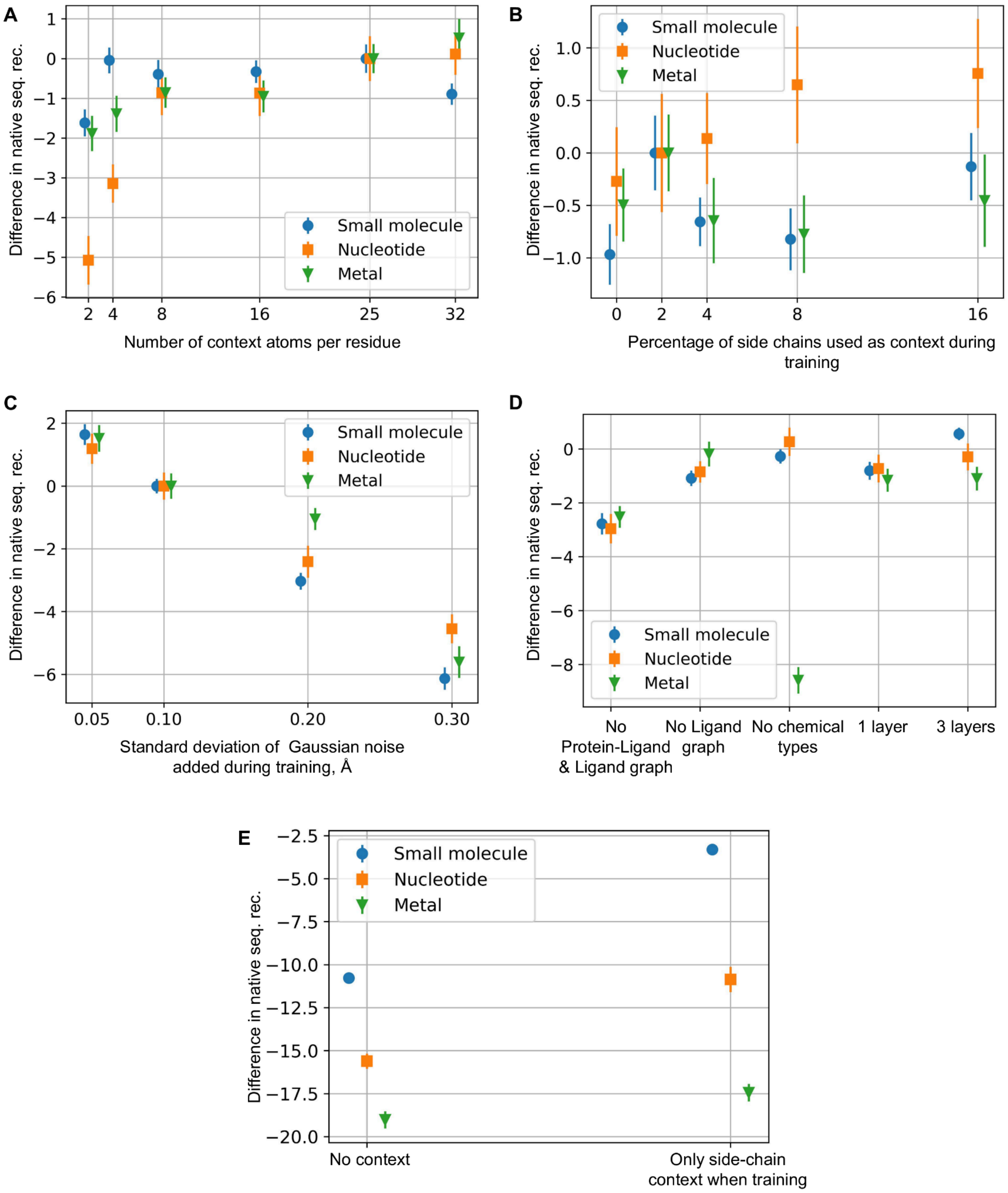

Figure S1. Different model variants.

(**A**) The difference in native sequence recovery for LigandMPNN models trained with a different number of context atoms per residue. The baseline model was trained with 25 atoms per residue. (**B**) The difference in native sequence recovery as a function of the percentage of sidechains used as context atoms during training. (**C**) Sensitivity of the sequence recovery to the magnitude of Gaussian noise added during training to protein & context atoms. (**D**) Sequence recovery changes for different ablations. For the "No Protein-Ligand & Ligand graph," chemical element types and geometry were used directly as node inputs for the protein graph. For the "No Ligand graph," we kept 2 MPNN layers of

Protein-Ligand only. For the "No chemical types," we passed zeros as chemical element types when training and during the inference. For the "1/3 layers", we used 1 or 3 instead of 2 baseline model Protein-Ligand & Ligand MPNN layers. (**E**) Comparing LigandMPNN with the model trained without any context (ProteinMPNN) and with the LigandMPNN model trained with only protein sidechain context. All figures are showing mean and standard deviation for 317 small molecule, 74 nucleotide, and 83 metal containing test proteins with sequence recovery reported as average over 10 sequences.

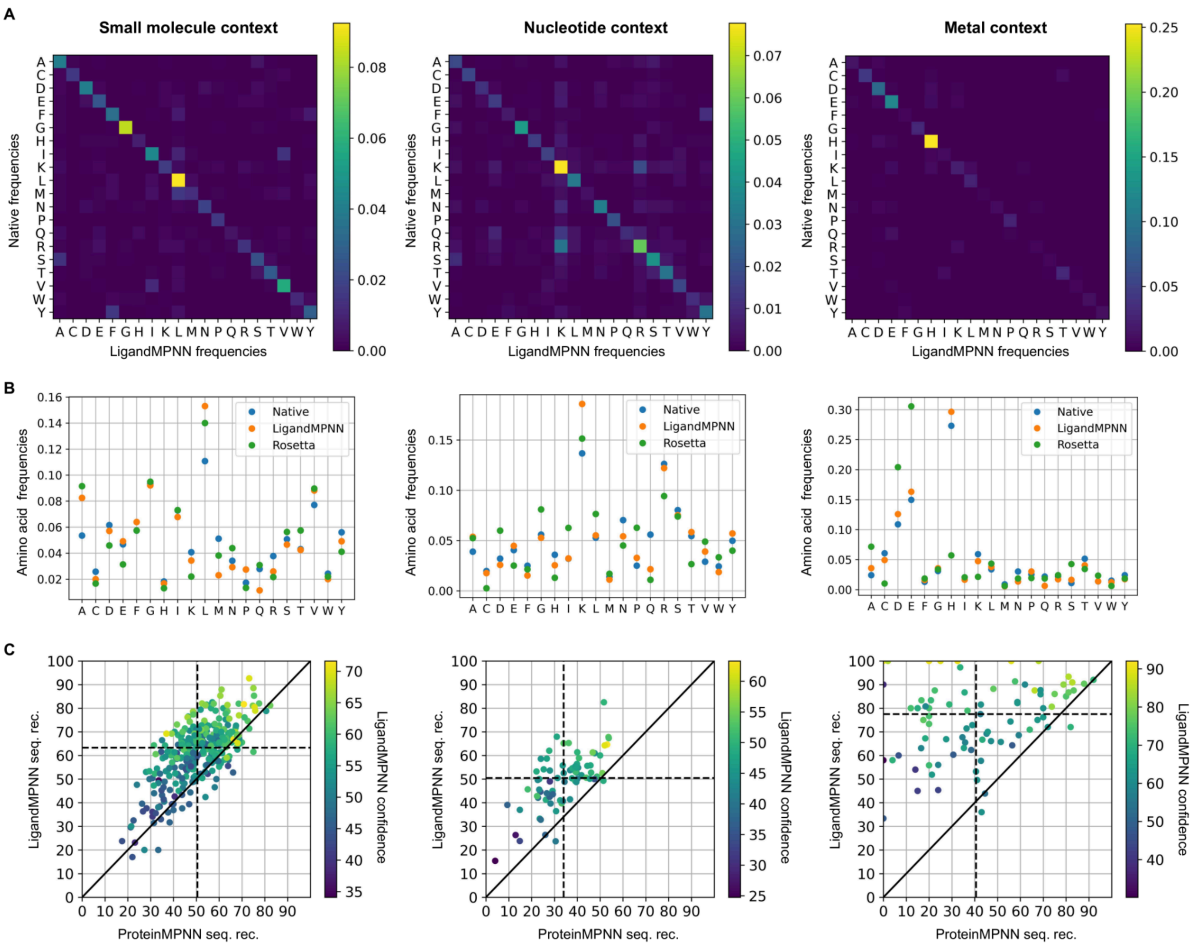

Figure S2. Sequence biases for residues within 5.0 Å from ligands.
(**A**) Confusion matrices for LigandMPNN. (**B**) Amino acid biases comparing native vs LigandMPNN vs Rosetta sequences. (**C**) Comparing ProteinMPNN vs LigandMPNN per protein basis. Color represents LigandMPNN confidence. Dashed lines show mean values.

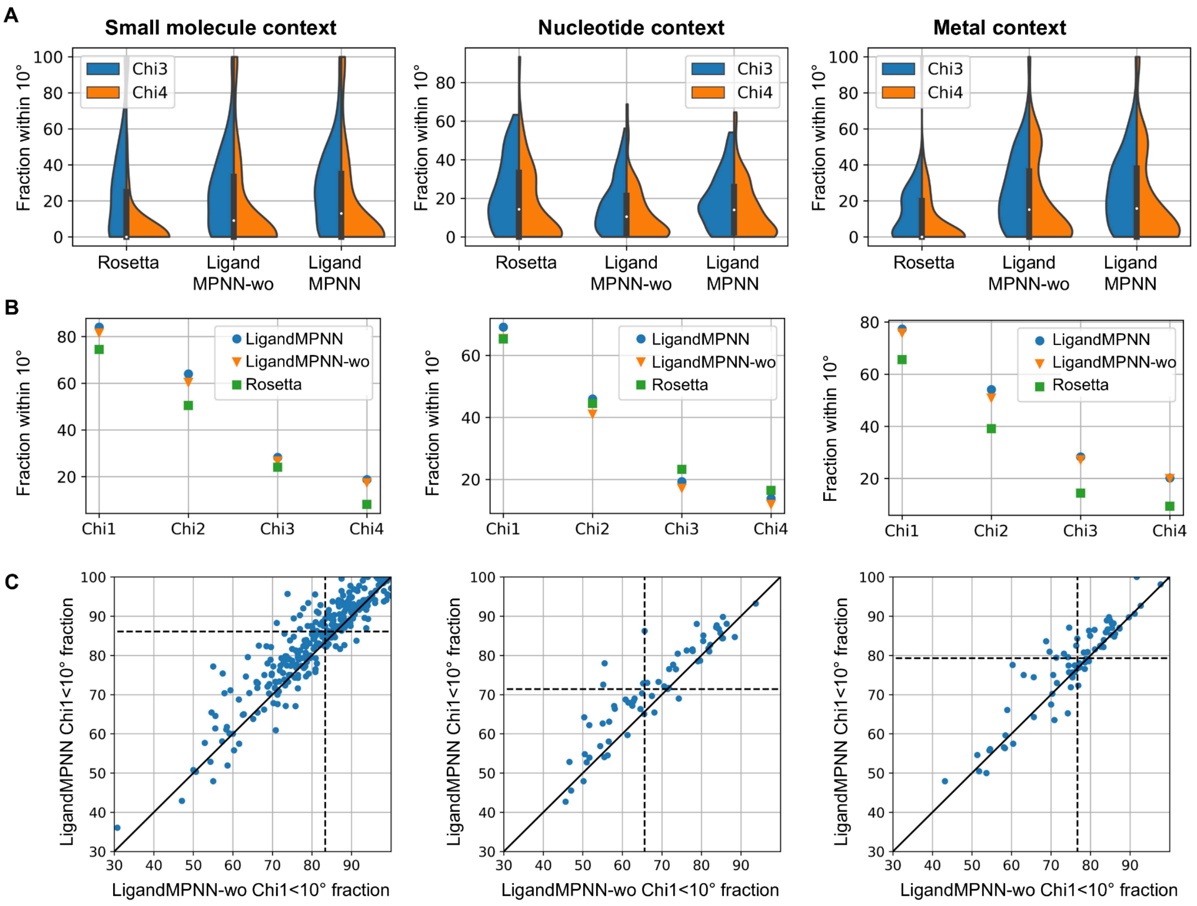

Figure S3. Sidechain chi angle recoveries for residues within 5.0 Å from ligands.

(**A**) Shows distributions for fractions within 10 degrees from a native conformation of chi3 and chi4 for Rosetta, LigandMPNN-wo, and LigandMPNN. (**B**) Mean chi recoveries for LigandMPNN, LigandMPNN-wo, and Rosetta. (**C**) Per protein comparison of chi1 recovery of LigandMPNN-wo vs LigandMPNN. Dashed lines show mean values. We reported average chi1 recovery over ten sidechain packing samples for one protein for 317 small molecule, 67 nucleotide, and 76 metal-containing test proteins.

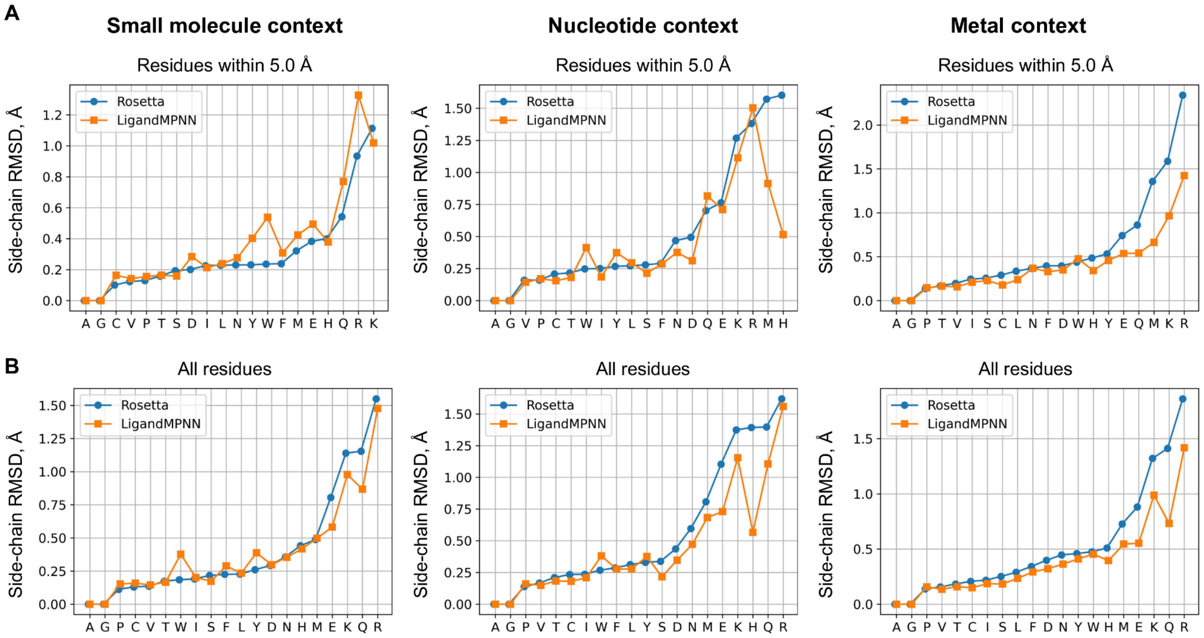

Figure S4. Median sidechain root-mean-square deviations (RMSD) per amino acid for Rosetta vs LigandMPNN.

(**A**) RMSDs over protein residues within 5.0 Å from the context atoms, (**B**) over all residues in the protein chain.

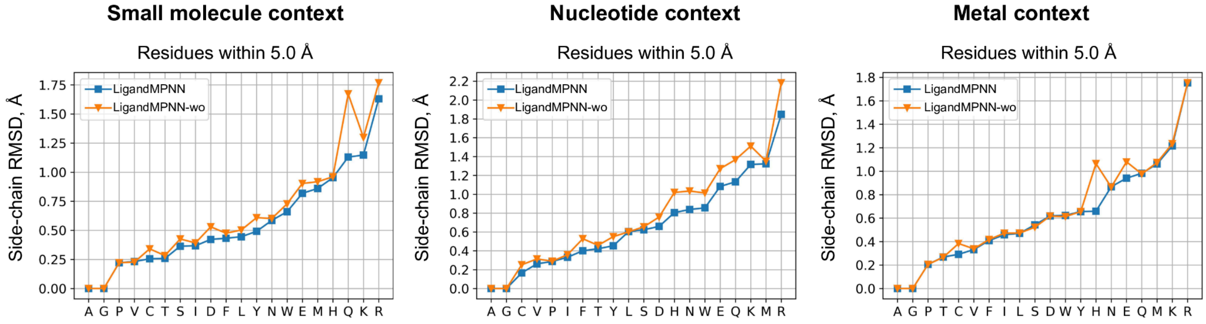

Figure S5. Comparing LigandMPNN vs LigandMPNN-wo for sidechain packing.

The biggest improvements in terms of sidechain root-mean-square deviation (RMSD) are obtained for glutamine (Q) in the small molecule dataset, for arginine (R) in the nucleotide dataset, and for histidine (H) in the metal context dataset.

Small molecule context benchmark PDB IDs

['1a28', '1bzc', '1drv', '1e3g', '1elb', '1elc', '1epo', '1f0r', '1g7f', '1g7g', '1gvw', '1gx8', '1i37', '1kav', '1kdk', '1kv1', '1l8g', '1lhu', '1lpg', '1nc1', '1nfx', '1nhz', '1nl9', '1nny', '1nwl', '1ony', '1pyn', '1qb1', '1qkt', '1qxk', '1r0p', '1sj0', '1sqn', '1v2n', '1xjd', '1xws', '1yc1', '1yqj', '1z95', '1zp8', '2ayr', '2b07', '2b4l', '2baj', '2bak', '2bal', '2bsm', '2cet', '2e2r', '2f6t', '2fdp', '2g94', '2hah', '2ihq', '2iwx', '2j2u', '2j34', '2j4i', '2j94', '2j95', '2o0u', '2oax', '2ojg', '2ojj', '2p4j', '2p7g', '2p7z', '2pog', '2qbp', '2qbq', '2qbs', '2qe4', '2qmg', '2uwl', '2uwo', '2uwp', '2v7a', '2vh0', '2vh6', '2vkm', '2vrj', '2vw5', '2vwc', '2w8y', '2wc3', '2web', '2wec', '2weq', '2wgj', '2wuf', '2wyg', '2wyj', '2xab', '2xb8', '2xda', '2xht', '2xj1', '2xj2', '2xjg', '2xjx', '2y7x', '2y7z', '2y80', '2y81', '2y82', '2ydw', '2yek', '2yel', '2yfe', '2yfx', '2yge', '2ygf', '2yi0', '2yi7', '2yix', '2zmm', '3acw', '3acx', '3b5r', '3b65', '3bgq', '3bgz', '3ckp', '3cow', '3coy', '3coz', '3d7z', '3d83', '3eax', '3ekr', '3fv1', '3fv2', '3fvk', '3gba', '3gbb', '3gcs', '3gcu', '3gy3', '3hek', '3i25', '3ioc', '3iph', '3iw6', '3k97', '3lpi', '3lpk', '3lxk', '3m35', '3myg', '3n76', '3nq3', '3nyx', '3o5x', '3o8p', '3pww', '3roc', '3tfn', '3u81', '3ueu', '3uev', '3uew', '3uex', '3vha', '3vhc', '3vhd', '3vje', '3vvy', '3vw1', '3vw2', '3wha', '3wz6', '3wz8', '3zc5', '3zm9', '3zze', '4a4v', '4a4w', '4a7i', '4ag8', '4ap7', '4b6o', '4b9k', '4cd0', '4cga', '4cmo', '4da5', '4e5w', '4e6d', '4e9u', '4ea2', '4egk', '4er1', '4fcq', '4ffs', '4flp', '4g8n', '4gny', '4gu6', '4hge', '4igt', '4k0y', '4k9y', '4kao', '4kcx', '4lyw', '4m0r', '4m12', '4m13', '4muf', '4nh8', '4nwc', '4o04', '4o05', '4o07', '4o09', '4o0b', '4p5z', '4pmm', '4pop', '4qev', '4qew', '4qyy', '4rfm', '4rwj', '4twp', '4uyf', '4v01', '4w9f', '4w9l', '4wa9', '4wkn', '4x6p', '4xip', '4xir', '4y79', '4ybk', '4ymb', '4yml', '4ynb', '4yth', '4z0k', '4zae', '5aa9', '5acy', '5d26', '5d3h', '5d3j', '5d3l', '5d3t', '5dlx', '5dqc', '5dwr', '5e74', '5egm', '5eng', '5eqp', '5eqy', '5er1', '5exm', '5exn', '5f9b', '5fto', '5fut', '5hcv', '5i3v', '5i3y', '5i9x', '5i9z', '5ie1', '5ih9', '5jq5', '5kz0', '5l2s', '5lli', '5lny', '5lsg', '5neb', '5nw1', '5nyh', '5op5', '5oq8', '5qqp', '5t19', '5tpx', '5v82', '5yfs', '5yft', '6c2r', '6cjr', '6cpw', '6dgq', '6dgr', '6dyu', '6dyv', '6el5', '6elo', '6elp', '6ey9', '6eyb', '6f1n', '6ge7', '6gf9', '6gfs', '6ghh', '6i61', '6i64', '6i67', '6md0', '6mh1', '6mh7', '6n7a', '6n8x', '6no9', '6nv7', '6nv9', '6olx', '6qi7']

Nucleotide context benchmark PDB IDs

['1a0a', '1am9', '1an4', '1b01', '1bc7', '1bc8', '1di2', '1ec6', '1hlo', '1hlv', '1i3j', '1pvi', '1qum', '1sfu', '1u3e', '1xpx', '1yo5', '1zx4', '2c5r', '2c62', '2nq9', '2o4a', '2p5l', '2xdb', '2ypb', '2zhg', '2zio', '3adl', '3bsu', '3fc3', '3g73', '3gna', '3gx4', '3lsr', '3mj0', '3mva', '3n7q', '3olt', '3vok', '3vwb', '3zp5', '4ato', '4bhm', '4bqa', '4e0p', '4nid', '4wal', '5cm3', '5haw', '5mht', '5vc9', '5w9s', '5ybd', '6bjv', '6dnw', '6fqr', '6gdr', '6kbs', '6lff', '6lmj', '6od4', '6wdz', '6x70', '6y93', '7bca', '7c0g', '7el3', '7jsa', '7ju3', '7kii', '7kij', '7mtl', '7z0u', '8dwm']

Metal context benchmark PDB IDs

['1dwh', '1e4m', '1e6s', '1e72', '1f35', '1fee', '1job', '1lqk', '1m5e', '1m5f', '1moj', '1mxy', '1mxz', '1my1', '1nki', '1qum', '1sgf', '1t31', '1u3e', '2bdh', '2bx2', '2cfv', '2e6c', '2nq9', '2nqj', '2nz6', '2ou7', '2vxx', '2zwn', '3bvx', '3cv5', '3f4v', '3f5l', '3fgg', '3hg9', '3hkn', '3hkt', '3i9z', '3k7r', '3l24', '3l7t', '3m7p', '3mi9', '3o1u', '3u92', '3u93', '3u94', '3won', '4aoj', '4dy1', '4hzt', '4i0f', '4i0j', '4i0z', '4i11', '4i12', '4jd1', '4naz', '4wd8', '4x68', '5f55', '5f56', '5fgs', '5hez', '5i4j', '5l70', '5vde', '6a4x', '6buu', '6cyt', '6iv2', '6lkp', '6lrd', '6wdz', '6x75', '7dnr', '7e34', '7kii', '7n7g', '7s7l', '7s7m', '7w5e', '7wb2']

Computational design and experimental methods to validate small-molecule binding

LigandMPNN redesign for rocuronium binding

A set of previously designed binders for the small-molecule rocuronium (*2*) was redesigned using LigandMPNN to assess the performance of the model of generating sequences relevant to ligand binding. The original set consisting of 2,119 was previously screened using yeast cell surface display and fluorescence-activated cell sorting (*2*) and yielded a few hits. We computationally redesigned the sequences of the binders using LigandMPNN with the context of the original binding pose and backbone structure. For each input, 8 sequences were generated with temperature 0.1, and the updated design models with the new sequence threaded were relaxed with RosettaFastRelax (*3*) before computational filtering. We selected the designs based on the following metrics; having at least one hydrogen bond to the ligand rocuronium, Rosetta ddg lower than -30.0, Rosetta contact molecular surface (*4*) higher than 200, AlphaFold2 prediction average plddt higher than 85.0, and AlphaFold2 prediction model CA-RMSD and SC-RMSD less than 1.5 Å and 2.5 Å, respectively, compared to the design model.

We ordered the sequences of the selected 189 as eblock gene fragments (IDT) to test binding experimentally. Retrospectively, the 189 redesigned sequences originated from the original 111 designs. Using the gene fragments, linearized pETCON3 vector, and chemically competent EBY100 yeast cells, we cloned and chemically transformed each design individually (*5*). The proteins were induced for expression and cell surface display in yeast media (C-Trp-Ura medium with 2% Glucose for cell growth and SGCAA medium with 0.2% Glucose for induction), and the ligand binding of each clone was analyzed using flow cytometry (Attune NxT, Thermo Fisher). The compensated PE signal indicated ligand binding

as we incubated the cells with biotinylated rocuronium (1uM), streptavidin conjugated to PE (Thermo Fisher, 1:20 dilution), and anti-c-myc antibody conjugated to FITC (Immunology Consultants Laboratory, 1:100 dilution).

We were able to rescue the ligand binding of one design with LigandMPNN. The original design didn't show any binding with the same analysis (Figure S6). Three gates were applied consecutively for analysis, and the final gate applied with a compensated FITC (expression) signal was used to generate the histogram in Figure 4A. The analysis was performed with FlowJo software v10.9.0.

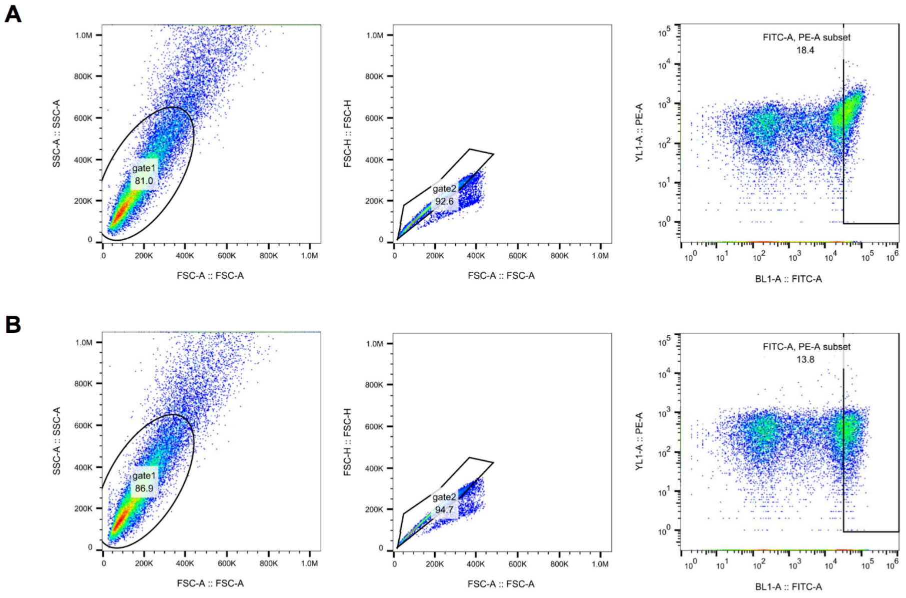

Figure S6. Flow cytometry analysis to test rocuronium binding.

Gates applied are shown with the compensated protein expression (FITC) and rocuronium binding signals (PE) for (**A**) LigandMPNN redesign and (**B**) the original design.

LigandMPNN redesign for Cholic acid (CHD) binding

The experimental validation of design power comparison between LigandMPNN and Rosetta was also performed on a validated micromolar CHD binder (Kd = 5.3 μM) (*6*). We hypothesized that, from an existing weak binder, generating several sequences using either LigandMPNN and Rosetta without changing the binding pose, the better sequence method would convey tighter binders from the generated sequences, while the less optimal one may break the original binding profile. From the crystal structure of the known CHD binder (8VEI), we used each of LigandMPNN and Rosetta to generate 1000 sequences. LigandMPNN was able to generate a diverse set of sequences based on the given CHD-binder complex; we randomly selected 105 of the 1000 sequences and ordered them as gene fragments from Twist Bioscience. Following previously reported protocols (*7*), we first tested these sequences for binding using yeast surface display. Using 5 μM FITC labeled CHD (CHD-FITC) and anti-cMyc-PE (Cell Signaling Technology, dilution 1:50), we found 21 sequences to be potential binders. Following the protocol reported previously (*7*), we purified

and tested the binding of 8/21 designs and found 6/8 designs to show better binding affinity ranging from 331 nM - 2.9 μM (Figure 4B), with significant improvements compared to the original binder with 5.3 μM binding affinity. For Rosetta, we ran a fix-backbone layered sequence design 1000 times (*8*). Within expectation, Rosetta converged to a single sequence after being calculated 1000 times because it is an energy-score-based design algorithm. Following the same procedure described above, we found the Rosetta-generated sequence lost the ligand binding ability both on yeast and using FP. Traditionally, when a weak binder is obtained, researchers first perform a site saturation mutagenesis experiment to identify beneficial mutations and then pool the beneficial mutations to generate a combinatorial library with size commonly ranging from 10^6-10^7; from the combinatorial library, variants with potentially improved affinity would be identified (*9*). This traditional method requires tedious labor work and often suffers from false positive binding signals originating from nonspecific binding. In comparison, LigandMPNN provided potentially better binder sequences within seconds, and through simple experiments, significantly tighter binders were obtained, implying the significant technological advance LigandMPNN brought to the binder design task.

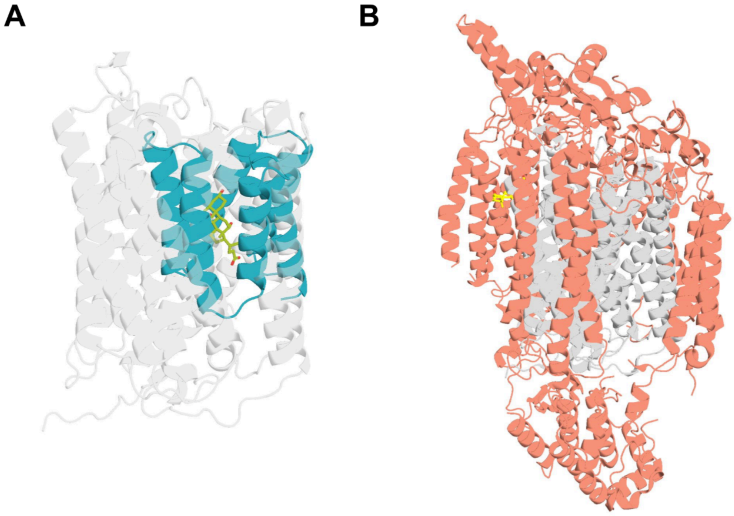

Figure S7. Comparing the designed cholic acid binder with the structurally closest cholic acid binding protein structure found in the PDB (6JY3).

(**A**) Structure of the designed cholic acid binder (teal, cholic acid in green) TMaligned to the closest cholic acid binding protein (grey, 6JY3 chain A, TM-score of 0.59). **(B)** Crystal structure of the complex structure of 6JY3 (chain A in grey, others in peach, cholic acid in yellow). Cholic acid (yellow) is bound in the interface of chain A (grey) and other chains (peach).

Crystal structure validation of designs generated with LigandMPNN

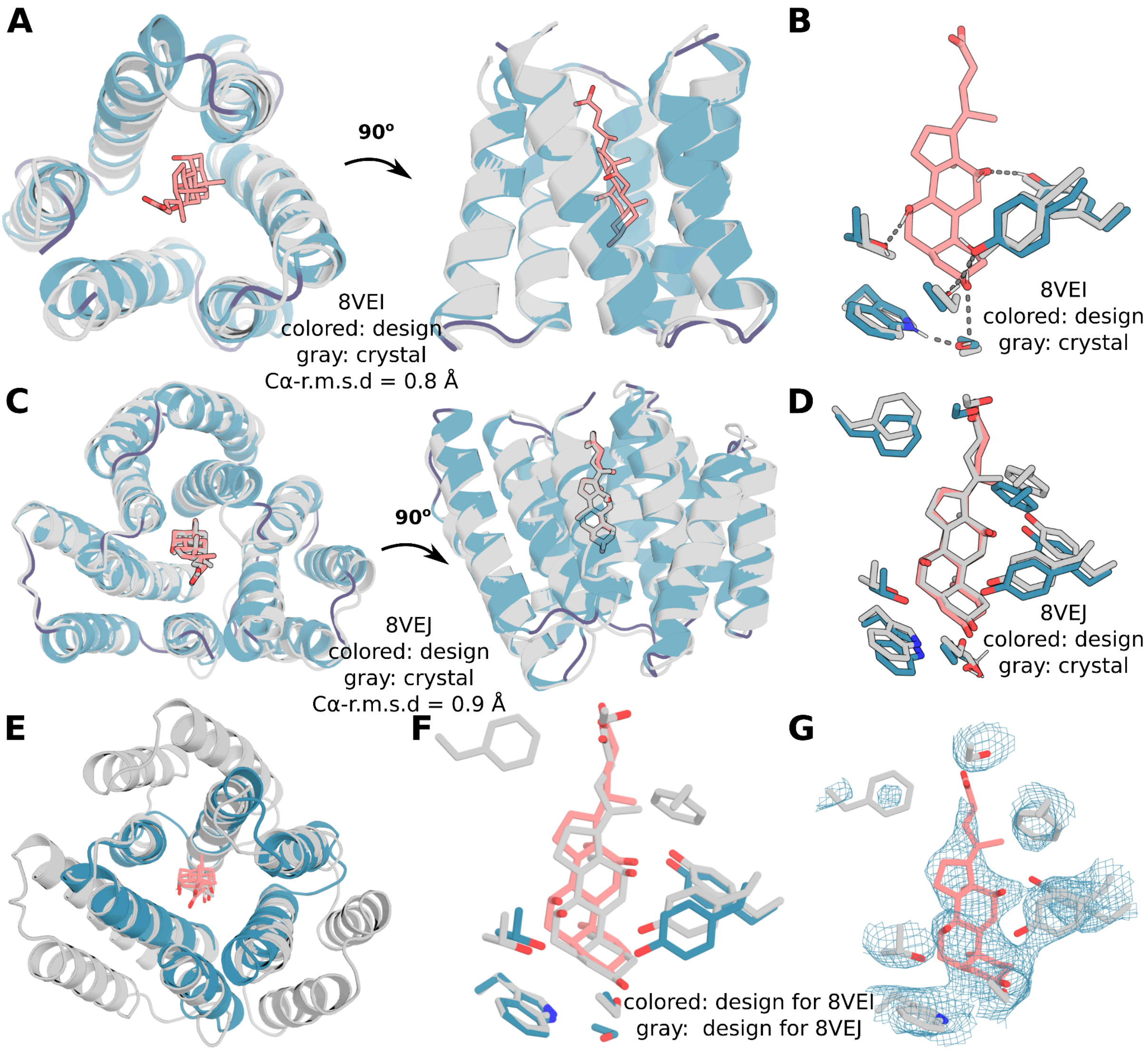

Figure S8. The design models and crystal structures of LigandMPNN-designed cholic acid binders (PDB ID: 8VEI and 8BEJ).

The crystal structure and the interface of LigandMPNN design cholic acid binder (**A, B**, PDB ID: 8VEI, resolution 2.0 Å) and a buttressed version of the same binder (**C, D**, PDB ID: 8VEJ, resolution 3.5 Å). (E, F) The two designs shared the same interface. (G) The interface 2mFo-DFc map at 1 σ level of the buttressed cholic acid binder. Also, see the crystal structure information on An et al. (7).

Multiple crystal structures were obtained to validate the designs generated using LigandMPNN, including two cholic acid binders (PDB IDs 8VEI and 8VEJ) (*7*), an apixaban binder (PDB IDs 8VEZ and 8VFQ. Two crystal structures solved for one design) (*2*), and a

DNA binder (PDB ID 8TAC) (*10*). The sequences of the designs were generated using LigandMPNN, and the crystal structure showed the residues LigandMPNN picked were largely well involved during ligand binding. We obtained two co-crystal structures of cholic acid binders, which share the same binding interfaces (Figure S8) (*7*). Both crystal structures closely resemble the designed structures (Figure S8 A, C), and the interfaces are well-represented in the crystal structures. LigandMPNN was successful in designing sequences for polar interactions and hydrogen bonding networks targeting the polar atoms of the cholic acid, and the structure of this interface was also accurately validated by crystal structures (Figure S8 B, D, G).

References

1. Dauparas, J., Anishchenko, I., Bennett, N., Bai, H., Ragotte, R. J., Milles, L. F., ... & Baker, D. (2022). Robust deep learning–based protein sequence design using ProteinMPNN. Science, 378(6615), 49-56.
2. Lee, G. R., Pellock, S. J., Norn, C., Tischer, D., Dauparas, J., Anishchenko, I., ... & Baker, D. (2023). Small-molecule binding and sensing with a designed protein family. bioRxiv, 2023-11.
3. Leaver-Fay, A., O'Meara, M. J., Tyka, M., Jacak, R., Song, Y., Kellogg, E. H., ... & Kuhlman, B. (2013). Scientific benchmarks for guiding macromolecular energy function improvement. In Methods in enzymology (Vol. 523, pp. 109-143). Academic Press.
4. Tinberg, C. E., Khare, S. D., Dou, J., Doyle, L., Nelson, J. W., Schena, A., ... & Baker, D. (2013). Computational design of ligand-binding proteins with high affinity and selectivity. Nature, 501(7466), 212-216.
5. Gietz, R. D., & Schiestl, R. H. (2007). High-efficiency yeast transformation using the LiAc/SS carrier DNA/PEG method. Nature protocols, 2(1), 31-34.
6. Kortemme, T. (2024). De novo protein design—From new structures to programmable functions. Cell, 187(3), 526-544.
7. An, L., Said, M., Tran, L., Majumder, S., Goreshnik, I., Lee, G. R., ... & Baker, D. (2024). Binding and sensing diverse small molecules using shape-complementary pseudocycles. Science, 385(6706), 276-282.
8. Tinberg, C. E., Khare, S. D., Dou, J., Doyle, L., Nelson, J. W., Schena, A., ... & Baker, D. (2013). Computational design of ligand-binding proteins with high affinity and selectivity. Nature, 501(7466), 212-216.

9. Dou, J., Vorobieva, A. A., Sheffler, W., Doyle, L. A., Park, H., Bick, M. J., ... & Baker, D. (2018). De novo design of a fluorescence-activating β-barrel. Nature, 561(7724), 485-491.
10. Glasscock, C. J., Pecoraro, R., McHugh, R., Doyle, L. A., Chen, W., Boivin, O., ... & Baker, D. (2023). Computational design of sequence-specific DNA-binding proteins. bioRxiv, 2023-09.
