# Supplementary material for: Atomic context-conditioned protein sequence design using LigandMPNN
Source: Nat Methods. 2025 Mar 28;22(4):717–23. doi: 10.1038/s41592-025-02626-1 (PMC11978504; doi:10.1038/s41592-025-02626-1)
Supplement: Supplementary file 2 — Reporting Summary [file 41592_2025_2626_MOESM2_ESM.pdf]

Corresponding author(s): DAVID BAKER, NMETH-A54598E

Last updated by author(s): Nov 7, 2024

Reporting Summary

Nature Portfolio wishes to improve the reproducibility of the work that we publish. This form provides structure for consistency and transparency in reporting. For further information on Nature Portfolio policies, see our Editorial Policies and the Editorial Policy Checklist.

Statistics

For all statistical analyses, confirm that the following items are present in the figure legend, table legend, main text, or Methods section.

n/a	Confirmed	
[ ]	[x]	The exact sample size (*n*) for each experimental group/condition, given as a discrete number and unit of measurement
[x]	[ ]	A statement on whether measurements were taken from distinct samples or whether the same sample was measured repeatedly
[x]	[ ]	The statistical test(s) used AND whether they are one- or two-sided<br>*Only common tests should be described solely by name; describe more complex techniques in the Methods section.*
[x]	[ ]	A description of all covariates tested
[x]	[ ]	A description of any assumptions or corrections, such as tests of normality and adjustment for multiple comparisons
[ ]	[x]	A full description of the statistical parameters including central tendency (e.g. means) or other basic estimates (e.g. regression coefficient) AND variation (e.g. standard deviation) or associated estimates of uncertainty (e.g. confidence intervals)
[x]	[ ]	For null hypothesis testing, the test statistic (e.g. *F*, *t*, *r*) with confidence intervals, effect sizes, degrees of freedom and *P* value noted<br>*Give P values as exact values whenever suitable.*
[x]	[ ]	For Bayesian analysis, information on the choice of priors and Markov chain Monte Carlo settings
[x]	[ ]	For hierarchical and complex designs, identification of the appropriate level for tests and full reporting of outcomes
[x]	[ ]	Estimates of effect sizes (e.g. Cohen's *d*, Pearson's *r*), indicating how they were calculated

Our web collection on statistics for biologists contains articles on many of the points above.

Software and code

Policy information about availability of computer code

Data collection	The neural network was developed with PyTorch 1.11.0 (https://pytorch.org/get-started/locally/), cuda 11.1 (https://developer.nvidia.com/cuda-11.1.0-download-archive), NumPy v1.21.5 (https://github.com/numpy/numpy), Matplotlib v3.5.1 (https://github.com/matplotlib/matplotlib), Python v3.9.12 (https://www.python.org/).<br><br>MMseqs2 version 13-45111+ds-2 (https://github.com/soedinglab/MMseqs2) was used to cluster PDB chains, and mmcif vesion 0.84 (https://pypi.org/project/mmcif/), rdkit version 2022.03.2 (https://github.com/rdkit/rdkit) was used to parse PDB files.
Data analysis	Data analysis used Python v3.9.12 (https://www.python.org/), Matplotlib v3.5.1 (https://github.com/matplotlib/matplotlib), NumPy v1.21.5 (https://github.com/numpy/numpy). Structure visualizations were created in Pymol v2.3.5 (https://github.com/schrodinger/pymol-open-source). Flow cytometry data was analyzed using the software FlowJo v10.9.0.

For manuscripts utilizing custom algorithms or software that are central to the research but not yet described in published literature, software must be made available to editors and reviewers. We strongly encourage code deposition in a community repository (e.g. GitHub). See the Nature Portfolio guidelines for submitting code & software for further information.

Data

Policy information about availability of data

All manuscripts must include a data availability statement. This statement should provide the following information, where applicable:

- Accession codes, unique identifiers, or web links for publicly available datasets
- A description of any restrictions on data availability
- For clinical datasets or third party data, please ensure that the statement adheres to our policy

All input data are freely available from public sources.

PDB structures used for training were obtained from RCSB (https://www.rcsb.org/docs/programmatic-access/file-download-services). The PDB ids used in the paper: 8VEI, 8BEJ, 8VEZ, 8VFQ, 8TAC, 6JY3, 2P7G, 1BC8, 1E4M.

Human research participants

Policy information about studies involving human research participants and Sex and Gender in Research.

Reporting on sex and gender	N/A
Population characteristics	N/A
Recruitment	N/A
Ethics oversight	N/A

Note that full information on the approval of the study protocol must also be provided in the manuscript.

Field-specific reporting

Please select the one below that is the best fit for your research. If you are not sure, read the appropriate sections before making your selection.

☒ Life sciences ☐ Behavioural & social sciences ☐ Ecological, evolutionary & environmental sciences

For a reference copy of the document with all sections, see nature.com/documents/nr-reporting-summary-flat.pdf

Life sciences study design

All studies must disclose on these points even when the disclosure is negative.

Sample size	No sample size was chosen; the method was evaluated on PDB chains not in the training set (subject to the exclusions noted below).
Data exclusions	The PDB dataset was filtered removing all entries with lower than 3.5 angstrom resolution, chain with too few resolved residues, biological units with more than 6000 residues. This set was also redundancy reduced by clustering chains using 30% sequence identity, 80% coverage using MMseqs2 version 13-45111+ds-2 (https://github.com/soedinglab/MMseqs2).<br><br>No sample was excluded from the experimental data analysis.
Replication	The biotinylated rocuronium binding experiment using yeast cell surface display and flow cytometry was replicated twice to validate the binding signal. The fluorescence polarization experiments were performed independently twice and all replications were successful.
Randomization	Randomization was not needed for the binding experiments.
Blinding	Blinding was not needed for the binding experiments.

Reporting for specific materials, systems and methods

We require information from authors about some types of materials, experimental systems and methods used in many studies. Here, indicate whether each material, system or method listed is relevant to your study. If you are not sure if a list item applies to your research, read the appropriate section before selecting a response.

Materials & experimental systems

n/a	Involved in the study
☐	☒ Antibodies
☒	☐ Eukaryotic cell lines
☒	☐ Palaeontology and archaeology
☒	☐ Animals and other organisms
☒	☐ Clinical data
☒	☐ Dual use research of concern

Methods

n/a	Involved in the study
☒	☐ ChIP-seq
☐	☒ Flow cytometry
☒	☐ MRI-based neuroimaging

Antibodies

Antibodies used

anti-cMyc-PE (Cell Signaling Technology, Myc-Tag (9B11) Mouse mAb (PE Conjugate) #3739) in 1:50 dilution, anti-cMyc-FITC (Immunology Consultants Laboratory, CMYC-45F) in 1:100 dilution, Streptavidin R-Phycoerythrin Conjugate (SAPE)

Validation

anti-cMyc-PE (https://www.cellsignal.com/products/antibody-conjugates/myc-tag-9b11-mouse-mab-pe-conjugate/3739): "Myc-Tag (9B11) Mouse mAb (PE Conjugate) detects exogenously expressed proteins containing the Myc epitope tag. This antibody recognizes the Myc tag fused to either the amino or carboxy terminus of targeted proteins in transfected cells. Myc-Tag (9B11) Mouse mAb (PE Conjugate) detects exogenously expressed Myc-tagged proteins in cells expressed under a CMV promoter. Expression under other promoters has not been evaluated. The antibody may cross-react with c-myc protein."

anti-cMyc-FITC (https://www.icllab.com/anti-c-myc-antibody-chicken-fitc-conjugated-cmyc-45f.html) "This antibody will react with EQKLISEEDL as determined by ELISA and IEP techniques. It is suitable for blotting, ELISA and IF applications. Optimal working dilutions should be determined experimentally by the investigator"

Flow Cytometry

Plots

Confirm that:

☒ The axis labels state the marker and fluorochrome used (e.g. CD4-FITC).

☒ The axis scales are clearly visible. Include numbers along axes only for bottom left plot of group (a 'group' is an analysis of identical markers).

☒ All plots are contour plots with outliers or pseudocolor plots.

☒ A numerical value for number of cells or percentage (with statistics) is provided.

Methodology

Sample preparation

EBY100 yeast strain was used to clone in gene fragments of the designed sequences with pETCON3 vector for cell surface display.

Instrument

Attune NxT Flow Cytometer (Thermo Fisher) was used.

Software

The provided operating software of Attune was used to collect data, and the software FlowJo was used for data anaylsis and visualization.

Cell population abundance

We collected data of at least 30,000 cells per analysis.

Gating strategy

We applied gates to exclude outliers using FSC-A/SSC-A, followed by a gating strategy using FSC-A/FSC-H to exclude doublets.

☒ Tick this box to confirm that a figure exemplifying the gating strategy is provided in the Supplementary Information.
